# Effect of cryopreservation on CD4+ T cell subsets in foreskin tissue

**DOI:** 10.1371/journal.pone.0297884

**Published:** 2024-03-01

**Authors:** Omar Almomani, James Nnamutete, Zhongtian Shao, Victoria Menya Biribawa, HenryRoger Ssemunywa, Annemarie Namuniina, Brenda Okech, Sofya Ulanova, David Zuanazzi, Cindy M. Liu, Aaron A. R. Tobian, Ronald M. Galiwango, Rupert Kaul, Jessica L. Prodger

**Affiliations:** 1 Department of Microbiology and Immunology, Western University, London, Canada; 2 Rakai Health Sciences Program, Kalisizo, Uganda; 3 UVRI-IAVI HIV Vaccine Program Limited, Entebbe, Uganda; 4 Department of Environmental and Occupational Health, Milken Institute School of Public Health, George Washington University, Washington, DC, United States of America; 5 Department of Pathology, Johns Hopkins University School of Medicine, Johns Hopkins University, Baltimore, Maryland, United States of America; 6 Departments of Medicine and Immunology, University of Toronto, Toronto, Canada; 7 University Health Network, Toronto, Canada; Uniformed Services University, UNITED STATES

## Abstract

Voluntary medical male circumcision (VMMC) reduces HIV acquisition by at least 60%, but the determinants of HIV susceptibility in foreskin tissues are incompletely understood. Flow cytometry is a powerful tool that helps us understand tissue immune defenses in mucosal tissue like the inner foreskin, but foreskin flow cytometry has only been validated using fresh tissue samples. This restricts immune analyses to timepoints immediately after surgical acquisition and hinders research in this area. We compared fresh analysis with whole tissue cryopreservation and later thawing and digestion to analyze CD4+ T cell populations relevant to HIV susceptibility (CCR5, CD25, CD127, CCR4, CXCR3, CCR6, CCR10, HLA-DR, and CD38). Eight foreskin samples from HIV-negative males aged >18 years were collected after VMMC. For each sample, half the foreskin was immediately cryopreserved for later digestion and flow cytometry analysis, while the remaining tissues were analyzed fresh. We demonstrate no significant impact of cryopreservation on CD4+ T cell expression of CD25, CCR4, CCR6, HLA-DR, CCR10, or CD127. Although expression levels of CCR5, CD38, and CXCR3 were increased after cryopreservation, the relative ranking of participants was retained. In conclusion, cryopreserved foreskin tissues may be suitable for subsequent digestion and flow cytometry phenotyping of HIV-susceptible T cell populations.

## Introduction

Voluntary medical male circumcision (VMMC) reduces HIV acquisition in heterosexual men by at least 60% [1–3], but the mechanism(s) through which foreskin removal mediates HIV protection is not fully understood [4]. The tremendous public health benefits that VMMC programs have provided in priority countries [5,6] highlight the need for further research. Furthermore, VMMC reduces the acquisition of other viral sexually transmitted infections (STIs), including HPV and HSV-2 [1,7,8], and not only profoundly alters the penile microbiome of the host but also reduces bacterial vaginosis in female sexual partner(s) [9].

Although additional research on the immune parameters of HIV acquisition in the foreskin is needed, efforts to study foreskin immune cell populations and other immune parameters have been limited by the relative difficulty in obtaining these tissues from adults. Obtaining entire foreskin tissues during VMMC is relatively straightforward, but it is generally not feasible to obtain foreskin biopsies, which are more easily obtained at other mucosal sites such as the ectocervix, vagina, or anorectum. Therefore many published studies of HIV target cells in foreskin tissue have performed analysis immediately after circumcision using fresh samples [10–14]. Cryopreservation of foreskin tissues collected from adult males undergoing VMMC could substantially expand future research, allowing researchers to nest more sophisticated immune analyses in specific participants.

Unfortunately, there are currently no data regarding the effect of cryopreservation on foreskin T cell recovery relevant to HIV susceptibility on foreskin derived CD4+ T cells. Previous work in ectocervical and colorectal tissues demonstrated that the cryopreservation of whole tissues gives a higher cell yield and more representative T cell function than the cryopreservation of post-digestion single-cell suspensions [15]. In response to cryopreservation, the relative shifts in cell populations may be accounted for by the upregulation of particular markers [16] or the preferential death of subsets more susceptible to mechanical stress accumulated by cryopreservation [17]. Given the importance of the foreskin in HIV acquisition [4,7,18–28], we sought to investigate how cryopreservation alters the characteristics of potential CD4+ T cell targets.

## Methods

### Participants and sample processing

**Fig 1:**
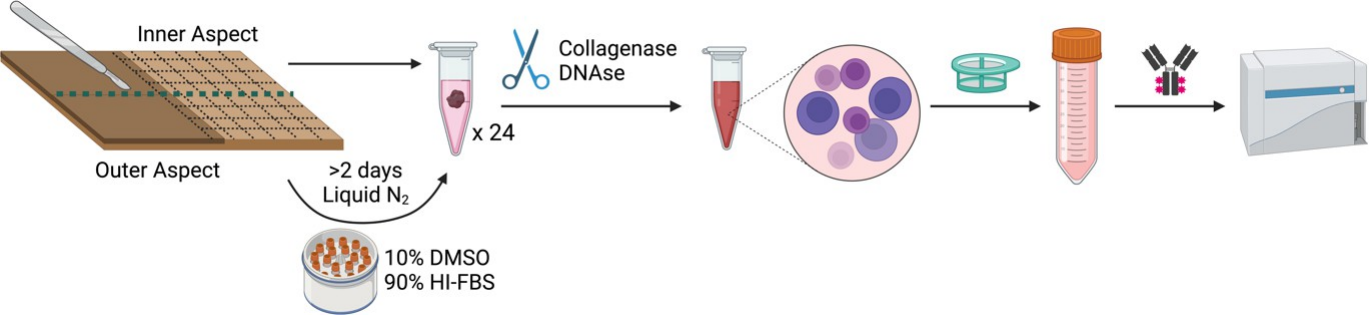
Schematic of tissue processing. Samples were divided equally, with one portion processed for flow cytometry immediately and one cryopreserved for 2–5 days before processing. Both samples were digested to a single cell suspension by mechanical and enzymatic digestion (collagenase) in the presence of DNAse. Cell suspensions were filtered to remove debris, stained with fluorescently labeled antibodies against markers of interest, and analyzed using an LSRII flow cytometer (HI-FBS: heat-inactivated FBS). Created with BioRender.com.

Participants were recruited from the VMMC program at Kisubi Hospital in Entebbe, Uganda. All participants were recruited in the months of August and September (2022), and participants provided informed consent verbally and in writing. Foreskin samples were collected from n = 8 HIV-negative adult males (18 years and older) with no clinically apparent genital signs or symptoms (discharge, ulcers, warts). Tissues were collected immediately after surgery and placed in a dry labeled 50 mL centrifuge tube without any transport media. Samples were without media for 15–30 minutes as they were transported from the site of VMMC to the research facility. Transport media was not used for two reasons. First, power supply is often inconsistent at sites of VMMC (including the areas surrounding Kisubi Hospital), and thus it is difficult to ensure consistent media refrigeration prior to VMMC. We therefore did not include media to ensure the methods reported in this study would be implementable in low-resources settings with inconsistent power supplies or lack of access to refrigeration. Moreover, media was avoided to maintain compatibility with studies where tissues will be later frozen for immunofluorescence (IF) microscopy. Our experience is that soaking tissues in media results in edema and tissue damage after freezing in Optimal Cutting Temperature (OCT) compound. Upon reaching the lab, the tissue was placed in a petri-dish and introduced to R10 media (RPMI with 10% HI-FBS, 10 mM HEPES buffer, 2 mM L-glutamine, 1 mM sodium pyruvate, 100 U/mL penicillin, 100 μg/mL streptomycin (all from Sigma)) to prevent drying of the tissue. The inner and outer aspects of the foreskin were identified, and the tissue was separated into two pieces containing equal quantities of inner and outer aspects (Fig 1). A 6 cm^2^ piece was dissected from each half using a scalpel, preferentially containing the inner aspect and augmenting with tissue from the outer aspect only when necessary (i.e., for smaller foreskins). Each 6 cm^2^ piece was further sectioned into 24 pieces, each measuring 0.25 cm^2^ squares that were either immediately processed for flow cytometry or cryopreserved for 2–5 days before processing.

### Cryopreservation and thawing of tissue

Each of the 24 tissue sections for cryopreservation was placed in a cryovial containing 1mL chilled cryopreservation media (10% DMSO, 90% HI-FBS, both Sigma). Cryovials were cooled to -80°C at 1°C /min using a freezing container (Mr. Frosty, Nalgene), then transferred to the vapor phase of liquid nitrogen for 2–5 days. Following cryopreservation, cryovials were removed from liquid nitrogen and agitated gently in a 37°C water bath until just thawed. Thawed tissues were transferred to 25mL of R10 for 10 minutes to allow DMSO to elute from the tissue.

### Cell isolation from foreskin tissues

Each tissue was placed in a 1.5mL snap-top tube (Eppendorf) containing 0.5mL of RPMI, and sterile surgical scissors were used to mechanically disrupt tissues as much as possible. RPMI with collagenase (Type I powder, ThermoFisher Scientific) and DNAse I (ThermoFisher Scientific) were added to each tube to a final concentration of 500U/mL and 125 units/μl, respectively. Tubes were placed on a shaker with a heating block (Eppendorf ThermoMixer) at 900rpm and 37°C for 40 minutes. After 40 minutes, the cell suspensions were pooled into a 50mL conical tube containing 3mL FBS. The resulting suspension was filtered through a 100μm filter (Cell Strainer, Fisher Scientific) and centrifuged for 6 minutes at 241g. The cell pellet was gently resuspended in 20mL R10, and the number of live (trypan blue exclusion, Sigma) mononuclear cells was estimated by light microscopy. Isolated cells were rested overnight in an incubator at 37°C and 5% CO_2_.

### Flow cytometry

**Fig 2:**
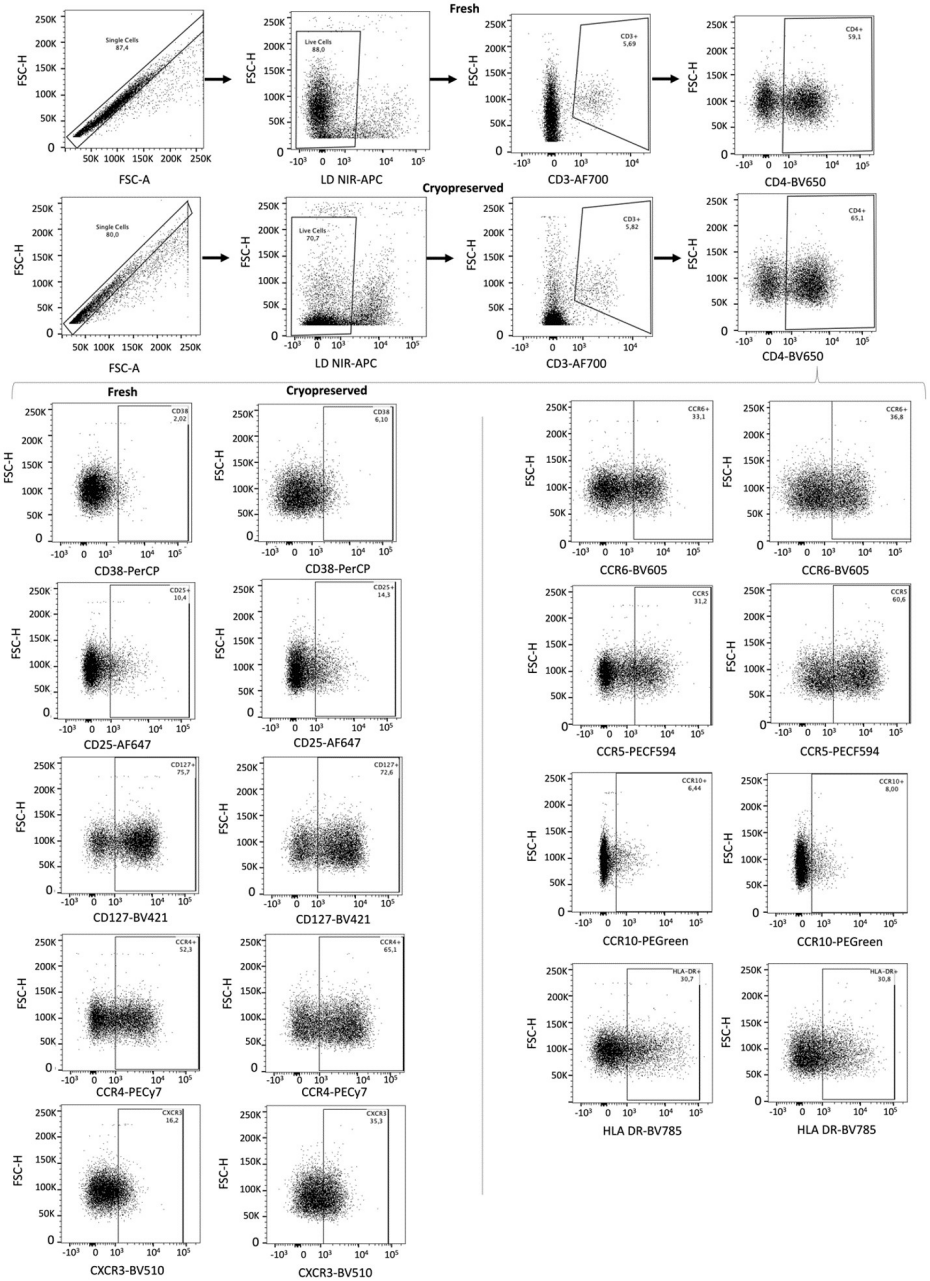
Gating strategy for identifying proportions of CD4+ markers in fresh vs. cryopreserved tissues. Doublet events were excluded based on height vs. area forward scatter, and dead cells were excluded. Live singlet events were then gated based on the positive expression of CD3, and the number of events in this gate was used to calculate the total T cells recovered. Total T cells were sub-gated on CD4+ events, and expression of all other phenotype markers was determined based on this population (shown for both tissues processed immediately after VMMC (fresh) and those processed after cryopreservation). Gates for each participant’s fresh and cryopreserved samples were identical, and FMO controls were used to assist with gating when populations were not clearly distinct.

Rested cells were washed and resuspended in 1mL PBS. Cells were stained first with LIVE/DEAD Fixable Near-IR Dead Cell Stain (Invitrogen), and then with the following fluorescently labeled anti-human antibodies (S1 Table) at 4°C for 20 minutes: CD3, CD4, CD38, HLA-DR, CD25, CD127, CD194 (CCR4), CD183 (CXCR3), CD196 (CCR6), CD195 (CCR5), and CCR10. Antibodies were chosen based on their ability to differentiate T cell populations relevant to HIV susceptibility [29–32]: Th1 (CXCR3+, CCR6-), Th2 (CCR4+, CCR6-), Th17 (CCR4+, CCR6+), Th22 (CCR10+, CCR4+, CCR6+), T regulatory cells (Treg, CD25+, CD127^LOW^); to identify activated T cells (HLA-DR and CD38); and expression of the HIV co-receptor essential for sexual transmission (CCR5) [33,34]. Cells were washed in PBS, fixed in 200mL of 2% formalin (Zayo-Sigma) for 20 minutes at 4°C, washed, and resuspended in 200mL of PBS. Flow cytometry data were acquired on an LSR II flow cytometer (BD Biosciences) using FACSDiva Software (BD Biosciences, version 6.0). Samples were run to completion, and the total number of live/dead-negative (live) and CD3+ events obtained for each sample was recorded as a measure of T cell recovery. Fluorescence Minus One (FMO) controls were run using cells from fresh foreskin tissues and PBMCs during panel development and antibody titration. FMOs were also run at the study midpoint (on PBMCs). Identical gates were applied to fresh and cryopreserved samples from the same participant. FMOs were used to guide gating when negative and positive populations were not readily discernable. The gating strategy is shown in Fig 2.

### Statistical analysis

Flow cytometry gating was performed using FlowJo software (version 10, FlowJo LLC). For each participant, gates were identical between the freshly digested portion of the foreskin and the cryopreserved counterpart. The proportion of live/CD3+ events positive for CD4 and the proportion of live/CD3+/CD4+ events positive for each remaining marker were compared between fresh and cryopreserved samples. Distributions of cell counts (for total T cells recovered) or proportional abundances (of markers as a % of live/CD3+/CD4+ cells) were compared using the Wilcoxon matched-pairs signed rank test. Statistical analyses were performed using Prism (v9, GraphPad).

### Ethics statement

All participants provided written informed consented to participate in this study. The study was reviewed and approved by research ethics boards at the Ugandan Virus Research Institute, the Uganda National Council for Science and Technology, the University of Toronto, and Western University.

## Results

**Fig 3:**
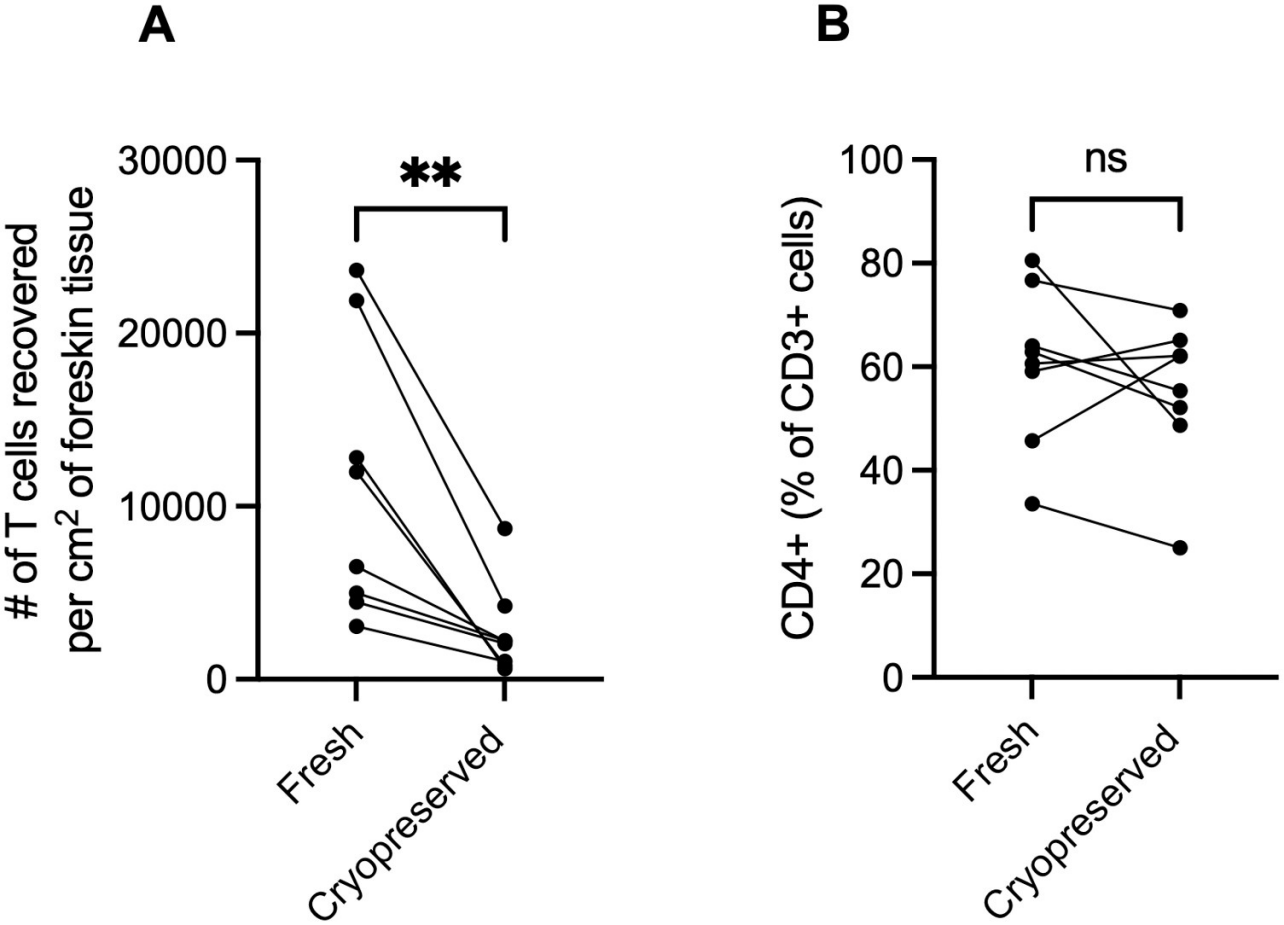
Effect of cryopreservation on the total number of T cells recovered per cm^2^ of foreskin tissue and relative abundance of T cells expressing CD4. (A) Total T cells recovered was defined as the total number of Live/Dead-, CD3+ events detected by flow cytometry when acquiring the complete sample generated from the digestion of 6cm^2^ of tissue. The total number of T cells recovered was higher in tissues processed immediately (fresh) than those processed after cryopreservation. (B) The relative abundance of T cells expressing CD4 did not differ between tissues processed immediately (fresh) compared to those processed after cryopreservation. Wilcoxon matched-pairs signed rank tests were used for both analyses (n = 8), *denotes p-value <0.05, ns (non-significant) denotes p-value >0.06, exact p-value listed when 0.06>p>0.05.

**Fig 4:**
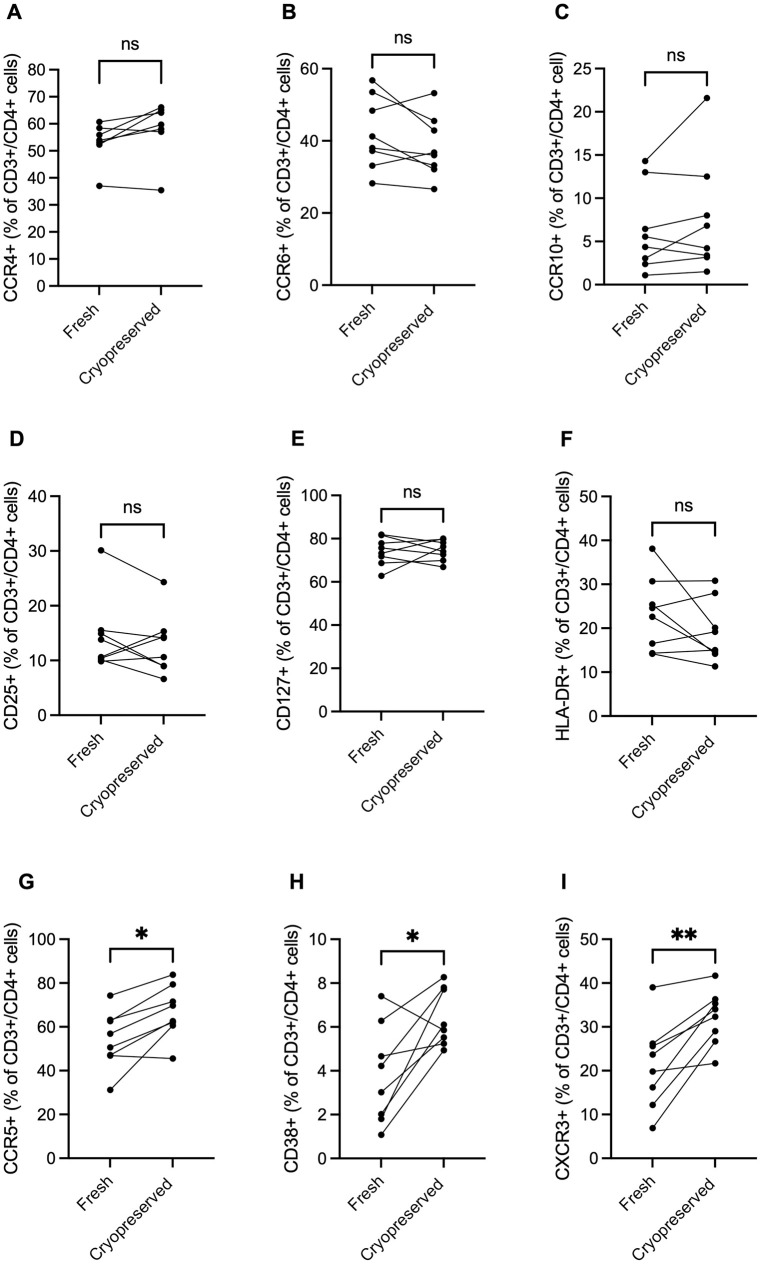
Effect of tissue cryopreservation on the expression of phenotype markers in CD4 T cells. There were no significant differences in the relative abundance of CD4 T cells expressing CCR4 (**A**), CCR6 (**B**), CCR10 (**C**), CD25 (**D**), CD127 (**E**), and HLA-DR (**F**) between tissues processed immediately (fresh) compared to those processed after cryopreservation. However, the relative abundance of CD4 T cells expressing CCR5 (**G**), CD38 (**H**), and CXCR3 (**I**) was significantly increased after cryopreservation. Wilcoxon matched-pairs sign rank test were used for all analyses (n = 8 pairs for all except for CCR4 where n = 7, Wilcoxon matched-pairs signed rank tests were used for both analyses (n = 8), *denotes p-value <0.05, ns (non-significant) denotes p-value >0.06, exact p-value listed when 0.06>p>0.05).

The total number of T cells recovered from fresh digestion of the foreskin was significantly higher than the number recovered from foreskin tissue that had been cryopreserved (median 9.3x10^3^ live CD3+ events vs. 2.1x10^3^, p <0.01, Fig 3A). Within the T cell population (CD3+), the relative abundance of T cells expressing CD4 did not differ between fresh and cryopreserved tissue (median 62% vs. 58%, p = 0.38, Fig 3B). One sample was insufficiently stained for CCR4 and thus did not contribute to the analysis of this marker (n = 7 for comparing %CCR4+ in fresh vs. cryopreserved). There were also no significant differences in the relative abundance of CD4 T cells positive for CCR4 (median 54% vs. 59%, p = 0.08), CCR6 (median 40% vs. 36%, p = 0.20, CCR10 (median 5.0% vs. 5.5%, p = 0.38), CD25 (median 12% vs. 12%, p = 0.31), CD127 (median 74% vs. 75%, p = 0.95), and HLA-DR (median 24% vs. 17%, p = 0.38, Fig 4A–4F). However, after cryopreservation, there were statistically significant increases in the relative abundance of CD4 T cells expressing CCR5 (median 54% vs. 66%, p = 0.02), CD38 (median 3.6% vs. 6.0%, p = 0.02), and CXCR3 (median 22% vs. 33%, p<0.01, Fig 4G–4I). For CCR5 and CD38, 7/8 cryopreserved samples (n = 8) exhibited increased proportions of CCR5 and CD38 expression, while all samples showed increased proportions of CXCR3 expression. However, the relative ranking of participants’ expression of these markers was relatively stable (e.g., participants who ranked among the highest expressers in fresh conditions also ranked among the highest levels after cryopreservation). Similar findings were observed when examining the proportion of total CD3+ cells (i.e., not limiting to CD3+/CD4+ cells, S1 Fig).

**Fig 5:**
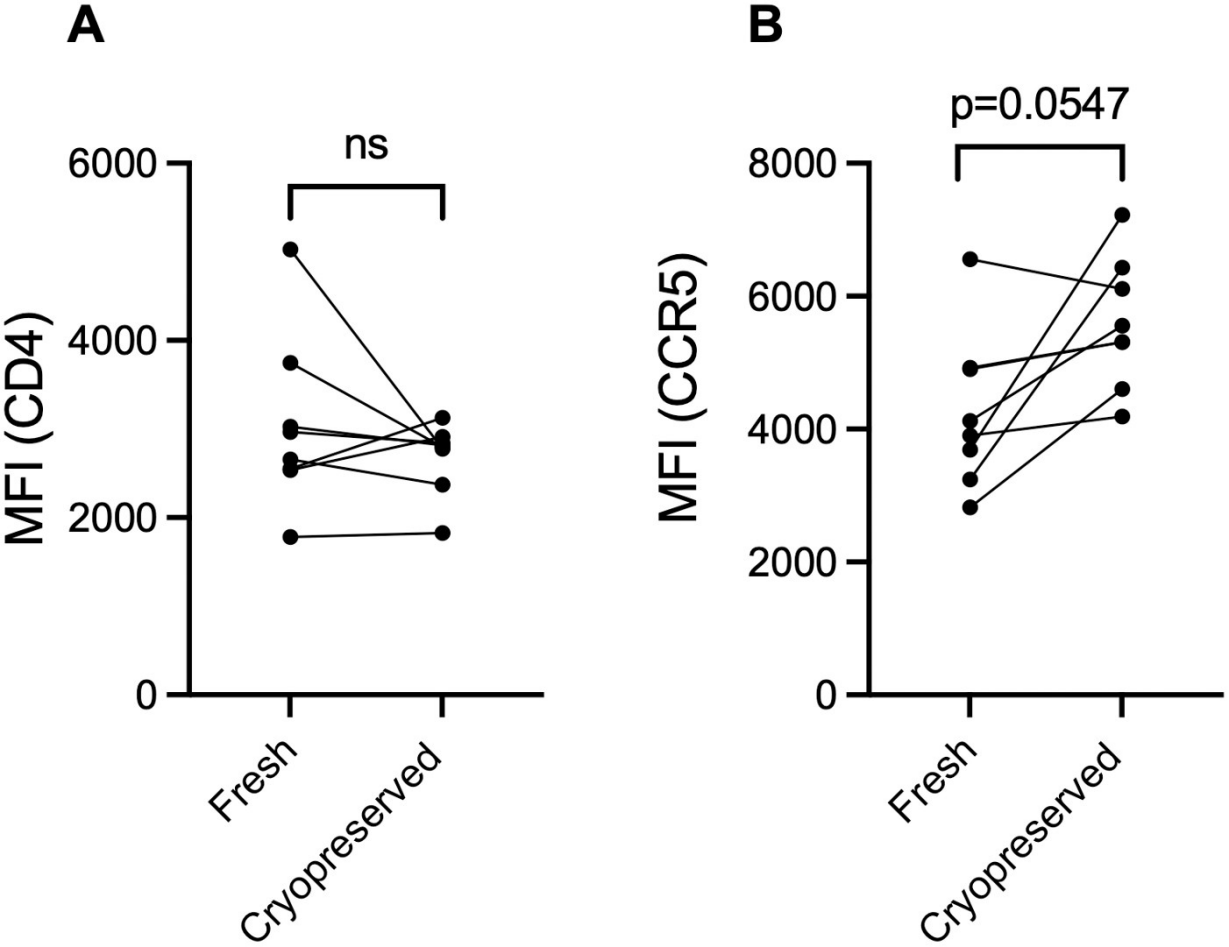
Effect of tissue cryopreservation on the Median Fluorescence Intensity (MFI) of CD4 and CCR5. MFI of (A) CD4+ or (B) CCR5+ cells was compared between tissues processed immediately (fresh) compared to those processed after cryopreservation. Wilcoxon matched-pairs sign rank test was used for this analysis (n = 8, *denotes p-value <0.05, ns (non-significant) denotes p-value >0.06, exact p-value listed when 0.06>p>0.05.

Due to their importance in HIV entry, we also examined if the per cell expression level of CD4 and CCR5 (i.e., Median Fluorescence Intensity, MFI) were altered by cryopreservation. We observed no change in CD4 MFI (Fig 5A); however, we did observe a non-significant increase in CCR5 MFI after cryopreservation (MFI 4013 vs. 5434, p = 0.0547).

## Discussion

To our knowledge, this is the first in-depth characterization of cryopreservation on foreskin tissue. The number of live T cells that can be isolated per cm^2^ of foreskin tissue was significantly reduced by cryopreservation. However, despite overall cell loss, adequate numbers were recovered for sub-gating on all 10 phenotypic markers in this study, and neither the relative abundance of T cells expressing CD4 nor the expression level of CD4 (MFI) differed significantly between matched fresh and cryopreserved samples. Among CD4 T cells, the relative abundance of cells expressing CCR4, CCR6, CCR10, CD25, CD127, and HLA-DR did not differ significantly between matched fresh and cryopreserved samples. In contrast, the relative abundance of CD4 T cells expressing CD38, CCR5, and CXCR3 was significantly increased in cells isolated from foreskin tissue samples that had been cryopreserved. We are unable to discern if this increase is due to selective death of cell populations that do not express these markers, or if these receptors are upregulated in response to cryopreservation [16]. However, our data suggest that the per cell level of CCR5 expression (MFI) may also increase with cryopreservation, although with the relatively small sample size this increase did not reach statistical significance (p = 0.0547). Despite the increased relative abundance of CD38, CCR5, and CXCR3, the rankings of matched participant samples for fresh vs. cryopreserved were largely maintained. Therefore, while cryopreservation will lead to an overestimation of the expression of CD38, CCR5, and CXCR3, the gross ranking of participants based on the expression of these markers will be maintained. This suggests that cryopreserved foreskin samples can still be used to compare these markers across study groups, so long as all samples being analyzed are cryopreserved.

The increased relative abundance of cells expressing CCR5 post-cryopreservation contrasts with a previous report in PBMCs. Costantini *et al*. performed an in-depth characterization of the effect of cryopreservation on PBMCs isolated from both HIV-infected and HIV-uninfected individuals, and observed a marked decrease in the abundance of cells expressing CCR5+ post-cryopreservation [35]. It is possible that cryopreservation of the tissue as a whole, as opposed to a cell suspension, followed by enzymatic digestion may account for the different findings in foreskin and PBMCs. Foreskin tissues were cryopreserved intact, as a previous study found that the cryopreservation of cervicovaginal and colorectal as tissue and then isolating cells upon thawing yielded a greater number of cells than cryopreservation as a single-cell suspension [15]. Like our study, they found that CD4 expression did not significantly differ between matched fresh and cryopreserved tissue samples. While that study did not explore most of the phenotypic markers we used, they defined T cells and other white blood cells using functional markers such as CD3, CD4, CD8, CD66b, CD33, CD13, and CD45. Ultimately, they report that T cell functions (i.e., cytokine production in response to stimulation) remained similar after cryopreservation. A limitation of this study is that tissues were maintained in liquid nitrogen for a relatively short time (2–5 days), and it is unknown if longer cryopreservation may induce further changes in cell populations. However, previous studies in PBMCs [36] have demonstrated that cell populations are not affected by the duration of cryopreservation, but instead by the number of freeze/thaw cycles that a sample is exposed to.

Despite the substantial loss of overall cell numbers, our results indicate that many CD4 T cells phenotypic markers relevant to HIV susceptibility are unchanged by cryopreservation. Three markers investigated (CD38, CXCR3, and CCR5) were consistently higher after cryopreservation, but the relative ranking of participants was generally maintained. Therefore, it may be possible to use cryopreserved tissues to examine differences between participants, but these data caution against comparing participants whose tissues were not cryopreserved.

## Supporting information

S1 Checklist Inclusivity in global research questionnaire. (DOCX)

S1 Data Raw data files for foreskin cryopreservation. (XLSX)

S1 Fig Effect of tissue cryopreservation on the expression of phenotype markers in CD3 T cells. (TIF)

S1 Table Details of antibodies used for flow cytometry. (TIF)
