# Supplementary figures and images for: Effect of cryopreservation on CD4+ T cell subsets in foreskin tissue
Source: PLoS One. 2024 Mar 1;19(3):e0297884. doi: 10.1371/journal.pone.0297884 (PMC10906856; doi:10.1371/journal.pone.0297884)

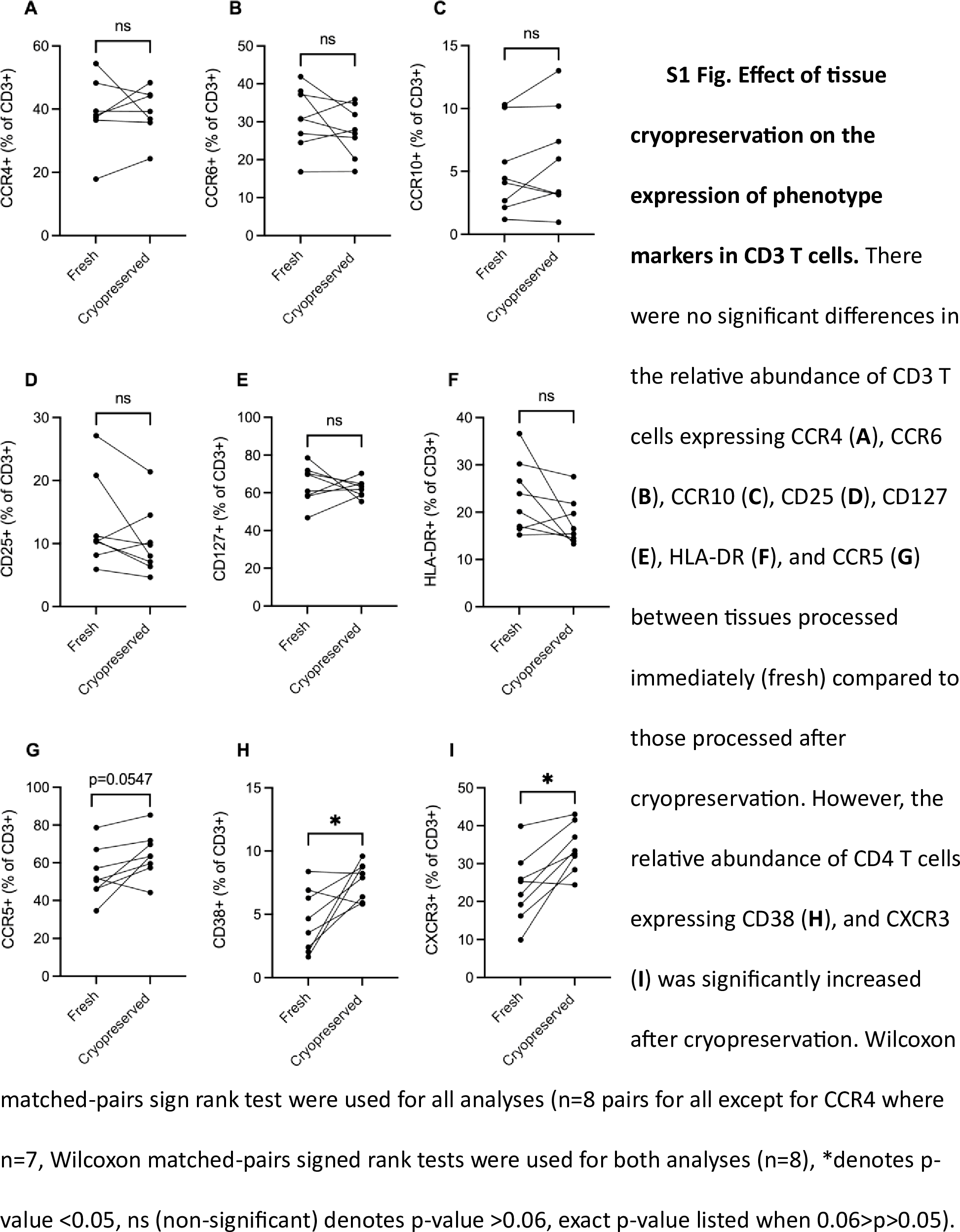

Supplement: S1 Fig — (TIF) [file pone.0297884.s003.tif]

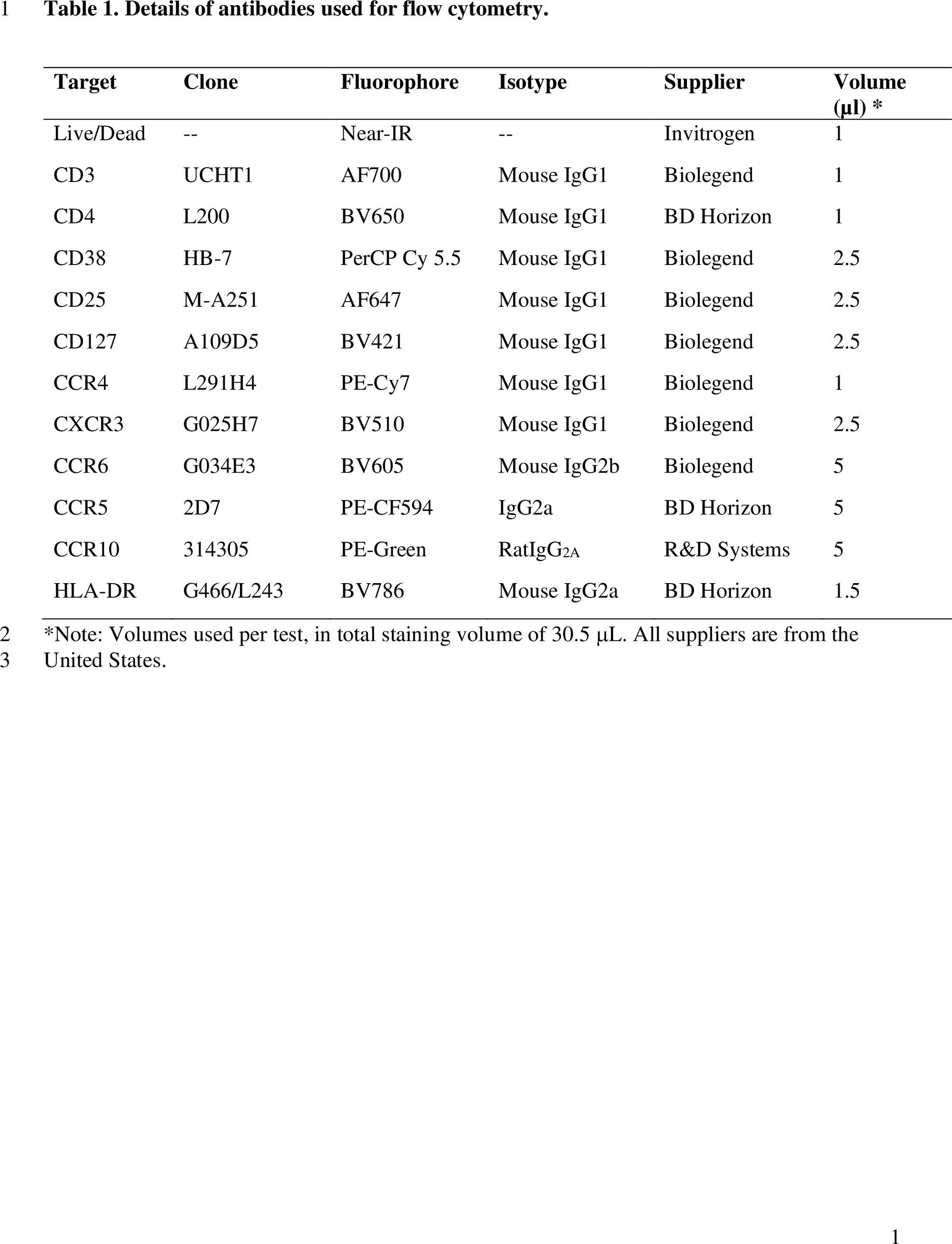

Supplement: S1 Table — (TIF) [file pone.0297884.s004.tif]
